# Exosomes Derived from microRNA-27a-3p Overexpressing Mesenchymal Stem Cells Inhibit the Progression of Liver Cancer through Suppression of Golgi Membrane Protein 1

**DOI:** 10.1155/2022/9748714

**Published:** 2022-12-07

**Authors:** Christian Cedric Bongolo, Erick Thokerunga, Qian Yan, Mohamed Bassirou Moukeila Yacouba, Chao Wang

**Affiliations:** ^1^Wuhan Sheba Precision Medical Technology Co. Ltd., Wuhan, 430022 Hubei, China; ^2^Department & Program of Clinical Laboratory Medicine, Center for Gene Diagnosis, Zhongnan Hospital of Wuhan University, Wuhan 43007, China; ^3^Department of Medical Laboratory Science, Faculty of Medicine, Mbarara University of Science and Technology, 1410 Mbarara, Uganda; ^4^Department of Anesthesiology, Zhongnan Hospital of Wuhan University, Wuhan 430071, China; ^5^Department of General Surgery, Clinical Research Center of Geriatric Diseases in Hubei Province, Tongji Hospital, Tongji Medical College, Huazhong University of Science and Technology, Wuhan 430030, China

## Abstract

Hepatocellular carcinoma (HCC) remains a significant health burden to date. Its early diagnosis and treatment are complicated by the lack of early diagnosis markers and multidrug resistance. microRNA regulation of HCC oncogenes are among the new diagnostic and therapeutic strategies being explored, although the mode of delivery of a therapeutic dose of the miRNA remains a challenge. In this study, we explored the use of exosomes from umbilical mesenchymal stem cells transfected with miR-27a-3p to interact with the oncogene GOLM1 in HCC and inhibit HCC progression both in vitro and in vivo. We first determined and compared the expression levels of miR-27a-3p in blood, various cell lines and tissues of HCC and their corresponding normal controls. We then employed bioinformatics analysis to determine the gene target for miR-27a-3p in HCC and later transfected upregulated miR-27a-3p in mesenchymal stem cells, and treated HCC cells with exosomes extracted from the transfected stem cells. We then created mouse models of HCC using balbc/nude mice and equally treated them with exosomes from miR-27a-3p transfected stem cells. The results showed that miR-27a-3p is downregulated in blood, cell lines, and tissues of HCC patients compared to normal controls. Exosomes from the miR-27a-3p transfected mesenchymal stem cells prevented HCC cell proliferation, invasion, and metastasis both in vitro and in vivo. Upregulation of miR-27a-3p prevented HCC through interacting with and downregulating GOLM1 as its target oncogene. In conclusion, miR-27a-3p is a potential therapeutic target for HCC acting through GOLM1.

## 1. Introduction

To date, hepatocellular carcinoma (HCC) remains a significant healthcare burden responsible for an enormous loss of life and livelihoods in both developed and developing countries [1, 2]. Global estimates indicate that approximately 841,000 new HCC cases and 782,000 HCC attributed deaths are recorded annually [3]. Viral hepatitis B and C, excessive alcohol intake, and aflatoxin-contaminated foodstuffs are the major risk factors for developing HCC [4]. Successful treatment of HCC cases is complicated by the lack of early diagnosis markers and the development of multidrug resistance, hence creating an urgent need for more novel and creative ways to diagnose and treat the cancer.

In an attempt to find new biomarkers for HCC diagnosis and treatment, Golgi membrane protein 1 (GP 73/GOLM1/GOLPH2)—a type II Golgi-membrane protein was discovered and shown to strongly promote HCC development and spread [5]. At the molecular level, GOLM1 induces HCC metastasis by interacting with and regulating epidermal growth factor receptor (EGFR) recycling at the cell surface, consequently promoting HCC cells' epithelial–mesenchymal transition [6]. It further facilitates the transport and secretion of matrix metallopeptidase 2 and 7 in HCC, another interaction that promotes HCC metastasis [7, 8]. This discovery has made GOLM1 a crucial therapeutic target for HCC.

GOLM1 expression is a factor of its messenger RNA translation, whose activities are in part controlled by microRNA (miRNAs). miRNA-559, miRNA-141-3p, and miRNA-27b-3p are some of the miRNAs known to control GOLM1 expression [9]. In relation to hepatic physiology, the miR-27a family, which among other things functions to maintain hepatic progenitor cells in their undifferentiated state has been shown to interact with GOLM1 [10]. miR-27a-5p is located on chromosome 19p13.12. It belongs to the miR-27a gene family, which is intergenic; located on chromosome 19, between miR24-2 and miR24a, and is transcribed from a noncoding gene [11]. It was found to have a potential binding site on the 3′-UTR region of GOLM1 [10]. These findings cast members of the miR-27a family, including miR-27a-3p that is the subject of this study, as potential regulators of GOLM1, as such, making them important targets for HCC therapy.

Although there is adequate experimental evidence to show that using miRNA mimics or antagomirs to correct specific alterations in miRNA expression normalizes aberrant gene regulation pathways and reverses cancer, the major challenge has always been how to effectively deliver miRNA to target cancer cells without being degraded or causing excessive toxicity [12]. In this study, we explore exosomes as a more efficient way to deliver the miR-27a-3p to HCC cells. Being of endogenous origin, exosomes have the unique ability to deliver its miRNA containing cargo to the target cells swiftly and with little or no toxicity at all [13]. We demonstrate that exosomes derived from miRNA-27a-3p overexpressing umbilical mesenchymal stem cells has the ability to interact with GOLM1 and inhibit the progression of HCC.

## 2. Materials and Methods

### 2.1. Blood Sample Collection

We collected whole blood samples from 90 HCC patients and 75 healthy control subjects who were attending Zhongnan Hospital of Wuhan University between March 2021 and July 2021. Only patients with a confirmed diagnosis of hepatocellular carcinoma and not yet started on any form of treatment were included as cases. Control subjects were healthy patients who had come for routine physical examination at the hospital. Informed consent was obtained from all subjects. The patients were classified into two subgroups; HCC patients (90 in total, 67 males and 23 females) with a mean age of 59.4 ± 11, and healthy control subjects (75 in total, 48 males and 27 females) with a mean age of 54.3 ± 13.

### 2.2. Hepatocellular Carcinoma Cell Line

Hepatocellular carcinoma cell lines HepG2, MHCC971, Hep3B, and Huh7 plus the normal liver cell line L02 were all obtained from Wuhan Procell Company Ltd., China, and cultured in high glucose DMEM media enriched with 10% fetal bovine serum (FBS) and 1% penicillin-streptomycin antibiotics.

### 2.3. Hepatocellular Carcinoma Tissue Sample Collection

We further collected 32 pairs of HCC and paracancerous tissue samples from HCC patients who underwent surgery. Samples were collected only from patients who were not yet initiated on treatment prior to surgery. The fresh samples were stored at -80°C until experiment time. Relevant clinical data of the patients were also collected. All patients provided informed consent prior to sample collection.

### 2.4. RNA Extraction and Reverse Transcription

Total RNA was extracted from plasma, cells, and tissue samples using RNzol-total RNA extraction kit (Cat: RP1001, Bioteke, Beijing, China), according to the manufacturers' instructions. Reverse transcription was performed using ReverTra Ace-qPCR RT kit from TOYOBO Co. Ltd. (Osaka, Japan). The reverse transcription conditions were as follows: 65°C for 5 min, 37°C for 5 min, and then 98°C for 15 min. The cDNA were temporarily kept at -20°C prior to use.

### 2.5. Real-Time Polymerase Chain Reaction Analysis

Quantitative real-time PCR was then performed using SYBR®Premix Ex Taq™ II real-time PCR kit (Takara, Japan) according to the manufacturer's instructions, on the Bio-Rad CFX PCR machine. A 20-*μ*L total reaction volume was used. The cycling conditions were as follows: initial denaturation fat 95°C for 5 min, 40 cycles of denaturation at 95°C for 30 sec, annealing at 61.4°C for 30 sec, and elongation at 72°C for 30 sec. Glyceraldehyde-3-phosphate dehydrogenase (GAPDH) was used as the endogenous control for data normalization. All reactions were carried out in duplicates. Primer sequences of the miR-27a-5p and GAPDH are provided in Table 1. The double delta Ct method was used to calculate gene expression.

### 2.6. Fluorescence In Situ Hybridization

To corroborate the qPCR results, fluorescence in situ hybridization (FISH) assay was conducted on the HCC and normal liver cell lines. A 5′ biotin-labeled probe (CTTCCAGTCGAGGATGTTTACA) designed to detect hsa-miR-27a-3p was obtained from Gema Biotech Ltd. Imaging was then conducted according to the FISH protocol as described by Arrigucci et al. [14].

### 2.7. Umbilical Mesenchymal Stem Cell Isolation and Identification

We isolated mesenchymal stem cells from fresh umbilical cords, collected from mothers who delivered from Zhongnan Hospital of Wuhan University. All the mothers consented to allow the use of their umbilical cords. The cords were rapidly washed with and rinsed twice in phosphate-buffered saline (PBS) containing sodium chloride and metronidazole. The cleaned cords were then cut to pieces, transferred to T75 culture bottles containing stem cell culture medium (Cyagen, Guangzhou, China), and incubated at 37°C in 5% CO_2_. Every three days from the date of initial culture, fresh media was changed. Approximately 10 days after the initial culture date, fibroblast-like cell colonies were observed. They were then passaged and cultured for 21 days in mesenchymal stem cell media (Gibco, GMP quality) with 10% fetal bovine serum (FBS) and 1% penicillin/streptomycin antibiotics (Sigma-Aldrich).

### 2.8. Exosome Extraction

When the MSCs in culture reached passage 2 (P2), plasmid transfection was conducted using Lipofectamine 2000 (Invitrogen, Carlsbad, CA, USA). The cells were first seeded in a 6-well plate at 1 × 10^6^ cell concentration and cultured to 80% confluence. One group was then transfected with hsa-miR-27a-3p encoding plasmids while the other group transfected with normal control plasmids. 48 hours after transfection, the culture media was removed and exosomes harvested from the supernatant using Bestbio exosome kit (Bestbio, Beijing, China) according to the manufacturer's instructions. Briefly, 20 mL of cell culture medium was collected and stored at 2-8°C. The medium was centrifuged at 3000 g for 15 minutes at 4°C and the supernatant was collected. The supernatant was then carefully transferred into another clean centrifuge tube, and again centrifuged at 1000 g for 20 min at 4°C. The sediment was discarded and supernatant collected and carefully moved into another clean centrifuge tube. 3 ml of reagent B (Bestbio exosome kit) was added to 12 ml of culture medium, capped tightly, mixed well and allowed to settle over night at 2-8°C. The next day, the mixture was centrifuged at 10000 g for 60 min at 4°C. The supernatant was carefully removed and the sediment was collected. The sediment containing the exosomes was then resuspended in 10 *μ*L of exosome preservation solution and stored at -80°C for downstream application. The protein concentration of the exosomes was measured using a BCA™ Protein Assay Kit (Beyotime, Shanghai, China), and the exosome proteins CD9 and CD63 was analyzed by western blot.

### 2.9. Transmission Electron Microscopy

#### 2.9.1. Nanoparticle Tracking Analysis

The sample centrifuged at 10,000 g was diluted 1: 1000 with PBS and 300 *μ*L loaded into the sample-holding chamber of an LM10 unit (Nanosight, Amesbury, UK). Three-30 second videos were recorded for each sample, and the data analyzed using the NTA 1.1 and 2.1 softwares (Nanosight). Basic control settings of the machine were maintained for sample analysis. Control beads of 100, 200, and 400 nm were obtained from Duke Scientific (Palo, Alto, CA). Shutter speeds for the beads were 30, 6, and 1 milliseconds for the 100, 200, and 400 nm beads, respectively, with zero camera gain. For the samples, shutter speeds were 30 or 15 milliseconds with camera gains ranging from 280 to 560. Analysis software was set as follows: detection threshold: 5-10; blur: auto; minimum particle size: 50 nm. All data were presented as average of two video recordings. Relative exosome concentration was calculated based on the dilution factor.

#### 2.9.2. Western Blot Assay

Exosomal protein was extracted using a cocktail of radio-immunoprecipitation assay (RIPA) buffer (Sangon Biotech Co., Ltd., China) and the protein inhibitor, phenylmethylsulfonyl fluoride (PMSF) solution (Thermo Fisher Scientific, Waltham, MA, USA). Bicinchoninic acid (BCA) assay (Beyotime, Shanghai, China) was performed to measure the protein concentration and 10 *μ*g of the protein separated by 10% SDS-PAGE gel electrophoresis. The separated proteins were then bloated onto polyvinylidene fluoride (PVDF) membranes (Merck KGaA), and blocked in 5% skimmed milk for 2 h at room temperature. The membrane was then incubated overnight with the following primary antibodies: CD63 (Cat: 67605-1-Ig, dilution: 1: 7000, Proteintech, China), CD9 (Cat: 60232-1-Ig, Dilution 1: 10,000, Proteintech, China), GOLM1 (Cat: HPA010638, dilution: 1: 5000, Sigma-Aldrich, Beijing, China), and *β*-actin (Cat: A5441, dilution: 1: 5000, Sigma-Aldrich, Beijing, China), all diluted as instructed by the manufacturer. They were then incubated in corresponding HRP secondary antibodies (Goat anti-mouse: Cat: 12-489, dilution: 1: 6000, goat anti-rabbit: Cat: 12-348, dilution: 1: 6000, all from Sigma-Aldrich, Beijing, China) and the bands on the PVDF membrane detected using the Chemiluminescent Substrate System (Thermo Fisher Scientific, USA).

#### 2.9.3. Cell Proliferation Assay

Twenty-four hours after transfection, the cells were seeded into 96-well plates (2000 cells/well) and cultured for 0 h, 24 h, 48 h, and 96 h. Then, 10 *μ*L of CCK8 solution was added to each well, and the plates incubated for 2 h at 37°C. Finally, the amount of formazan dye generated by cellular dehydrogenase activity was measured for absorbance at 450 nm by spectrophotometer (EnSpire, PerkinElmer, USA). The optical density values of each well were recorded as the measure of HCC cell proliferation.

#### 2.9.4. Transwell Assay

To verify cell migration, Transwell assay was conducted. Following transfection and 24 h incubation, cells were starved of FBS for 6 hours, then placed into the upper chamber (20,000 cells/chamber) of Transwell assay plates (Corning, USA), and 200 *μ*L of DMEM containing 10% FBS was added. After 24 h, the medium was removed and the cells that migrated to the lower chamber of the plate were fixed in 4% formaldehyde solution, stained with crystal violet for 20 minutes, and counted under light microscope.

#### 2.9.5. Flow Cytometry

Hepatocellular carcinoma cells were subcultured and treated with previously extracted umbilical MSC exosomes for 48 hours. They were then removed, fixed for 24 hours using 70% ethanol at 4°C, and resuspended in a solution containing phosphate buffered saline (PBS) and propidium iodide stain (Cat: P4170, Sigma-Aldrich, Beijing, China). The mixture was kept in the dark for 30 minutes, and then cell cycle analyzed using flow cytometry (EPICS XL, Beckman Coulter, USA).

#### 2.9.6. Bioinformatics Analysis

Bioinformatics analysis was conducted to search for genes that are significantly associated with miR-27a-3p. The bioinformatics tools Targetscan: https://www.targetscan.org/vert_80/, Diana tools: http://diana.imis.athena-innovation.gr/DianaTools/index.php, and miRBase: https://www.mirbase.org/ were used to analyze publicly available data. To narrow down number of hits and improve the probability of correct hits, search results were narrowed to only genes with 95% and above likelihoods of correct hits. All the predicted genes by the three tools were then put in the Bioinformatics and Evolutionary Genomics (BEG) software: http://bioinformatics.psb.ugent.be/webtools/Venn/ and a Venn diagram was generated to determine which genes were in the intersection of all three tools. Four highly potential miR-27a-3p binding genes (FBW7, PLK2, GRB2, and GOLM1) were generated.

#### 2.9.7. Animal Experiments

Eleven balbc/nude mice were acquired at 6 weeks old and kept in the laboratory for 2 weeks to acclimatize to the new environment. The mice were used at 8 weeks old to create HCC models for exosome challenge. All experiments were strictly conducted following the protocol approved by the Animal Care and Use Committee of Huazhong University of Science and Technology. The starting weight of the mice was 21-23 g. They were kept under the following conditions: a 14-hour light and 10-hour dark cycle, and temperatures of 18-23°C and 40-60% humidity. They were fed on laboratory pellets and provided adequate clean water. HepG2 cells were propagated and used to inject the mice and establish HCC models. After the establishment of the tumor models, the mice were divided into two groups; one group of 5 mice were injected with exosomes extracted from miR-27a-3p transfected MSCs, while the other group of 6 mice were injected with exosomes from MSCs transfected with normal control. The mice were observed for 21 days and results were recorded. To demonstrate the effect of miR-27a-3p on HCC metastasis, 6 balbc/nude mice were acquired and kept as before. They were divided into two groups of 3 mice each. The mice were operated under anesthesia and cultured HepG2 cells delivered directly into the liver. In the first group, exosomes extracted from the MSCs transfected with miR-27a-3p mimics were administered directly to the liver; while in the second group, MSCs transfected with normal control was administered. The mice were followed for three weeks, sacrificed, and liver tissues collected for HE staining, Masson's staining, and immunohistochemistry.

#### 2.9.8. Histological Examination and Immunohistochemistry

Mouse liver tissues collected after the HCC model creation and exomes treatments were stained with hematoxylin and eosin (H&E) and Masson's trichrome stain as described previously [15, 16] to demonstrate HCC metastasis. After histological examination, the expression of GOLM1 and the cancer proliferation marker (Ki-67) were performed by immunohistochemistry. In brief, the liver tissues were embedded in paraffin wax, then the paraffin removed, and the tissues rehydrated. They were then incubated for 30 minutes at room temperature in 5% normal blocking serum and stained with anti-GOLM1 antibody and the proliferation marker (Ki-67) antibody overnight at 4°C. The slides were then incubated with an appropriate secondary antibody for 60 min at room temperature, and the staining was examined. Staining intensity and proportions were viewed, and expression scores were calculated.

### 2.10. Statistical Analysis

All the experimental data were presented as mean ± SD of three independent experiments. The data were analyzed using the unpaired Student's *t*-test for difference between two independent groups and the one-way ANOVA for difference among multiple groups with Bonferroni and Dunnett's post hoc test, unless otherwise stated. Statistical analyses were conducted using GraphPad Prism version 8.2 (GraphPad Software Inc., USA). *P* < 0.05 was considered to be statistically significant.

## 3. Results

### 3.1. Expression of miR-27a-3p Was Downregulated in Hepatocellular Carcinoma Plasma, Cells, and Tissues

**Figure 1:**
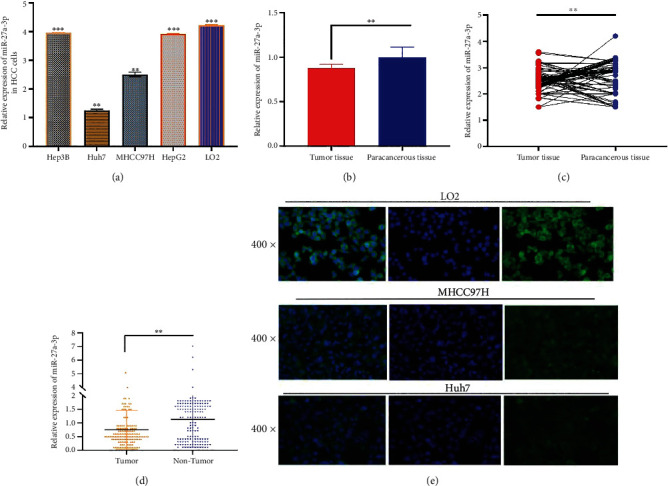
(a) Relative expression of miR-27a-3p in HCC and normal liver cell lines showing downregulated expression in HCC cell lines (Hep3B, Huh7, MHCC97H, and HepG2) compared to the normal liver cell line (L02). (b, c) Relative expression of miR-27a-3p in HCC tumor tissues (*n* = 32) and paracancerous tissues (*n* = 32) showing downregulated expression in the former compared to the latter. (d) Relative expression of miR-27a-3p in the blood of HCC patients (*n* = 90) and health controls (*n* = 75). Expression is downregulated in HCC patients compared to the healthy controls. Data were expressed as mean ± SD; ^∗∗^*P* < 0.01, ^∗∗∗^*P* < 0.001. (e) Relative expression of miR-27a-3p in HCC cell lines by fluorescence in situ hybridization analysis. Fluorescence intensity is higher in MHCC97H and HuH7 cells compared to L02.

As a first step, the expression level of miR-27a-3p was assessed in the plasma of 90 HCC patients/75 healthy controls, HCC/normal liver cell lines, and HCC tumor tissues/paracancerous tissues as controls. Real-time qRT-PCR and fluorescence in situ hybridization (FISH) assays were used. All experiments were conducted in duplicate. The qPCR results showed that the expression levels of miR-27a-3p in the HCC cell lines were significantly lower than those in the normal liver cells, and the same result was observed in the HCC tumor tissues vis-à-vis the paracancerous tissues, *P* < 0.007^∗∗^ (Figures 1(a)–1(c)). Similarly, the plasma levels of miR-27a-3p in HCC patients were significantly lower compared with healthy subjects, *P* < 0.001^∗∗^; (Figure 1(d)). Expressions of miR-27a-3p in the cell lines were further confirmed using fluorescence in situ hybridization (FISH), and the fluorescence intensity was very weak in the HCC cell lines compared to the normal liver cells, L02. (Figure 1(e)).

### 3.2. miR-27a-3p Is Correlated with some Clinical Variables of Hepatocellular Carcinoma Patients

We further assessed the relationship between miR-27a-3p and the HCC patients' clinical parameters to look for a clinically significant associations. As indicated in Tables 2 and 3, miR-27a-3p expression levels significantly correlated with tumor differentiation (*r* = 0.249, *P* = 0.039^∗^), tumor size (*r* = 0.258, *P* = 0.024^∗^), and TNM stage (*r* = 0.287, *P* = 0.015^∗^). However, no correlations were found regarding gender, age, smoking, alcoholism, cirrhosis, alpha-fetoprotein (AFP), hepatitis B virus-DNA, and other biochemical indices.

### 3.3. miR-27a-3p Is Significantly Correlated with GOLM1 in Hepatocellular Carcinoma Cells

**Figure 2:**
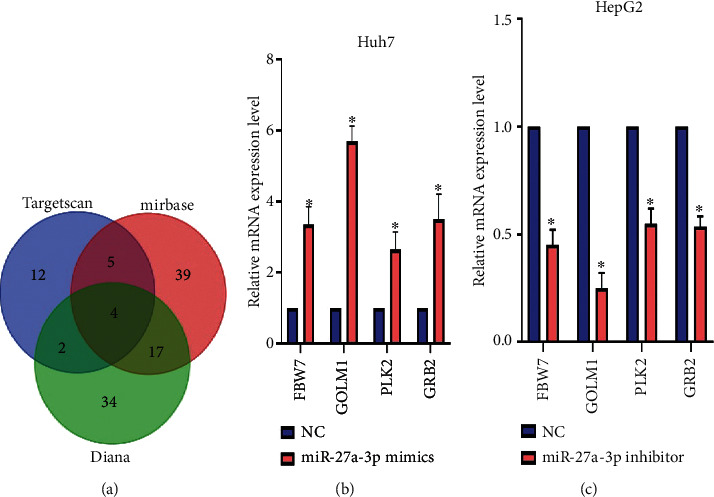
Bioinformatics results of potential miR-27a-3p binding genes. (a) Venn diagram showing most highly likely gene targets in the middle of the three circles. (b, c) Most likely gene target after experimental proof. Overexpression of miR-27a-3p in Huh7 cells downregulates GOLM1 and vice versa in HepG2 cells proving that it is the most likely gene target for miR-27a-3p. Data were expressed as mean ± SD; ^∗^*P* < 0.05.

We then embarked on a search for genes that are significantly associated with miR-27a-3p using bioinformatics analysis. We conducted searches using Targetscan, Diana tools, and miRBase. The search results were analyzed using Bioinformatics and Evolutionary Genomics (BEG) software and a Venn diagram was generated (Figure 2(a)). Four highly potential miR-27a-3p binding genes (FBW7, PLK2, GRB2, and GOLM1) were generated. To determine which of the four genes was most correlated with miR-27a-3p, Huh7, and HepG2 cells were chosen for miRNA mimics (overexpression) and antagomirs (knockdown) experiments, respectively. As presented in Figures 2(b) and 2(c), GOLM1 was the gene most correlated with miR-27a-3p.

### 3.4. Golgi Membrane Protein 1 Significantly Upregulated in Hepatocellular Carcinoma

**Figure 3:**
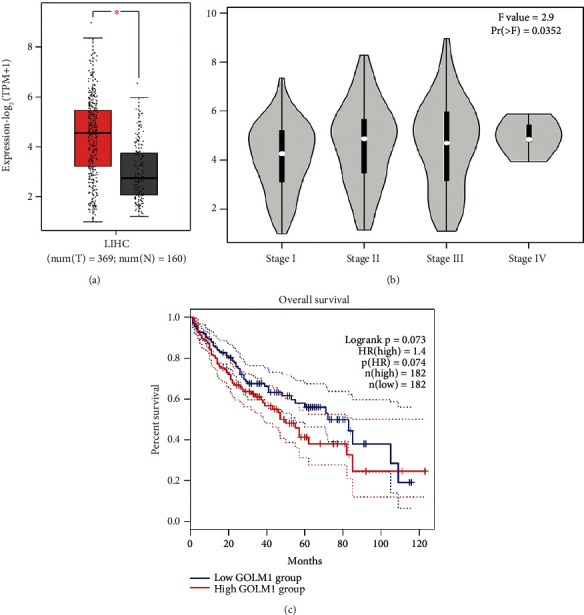
Bioinformatics analysis results in GEPIA2. (a) GOLM1 is significantly upregulated in HCC samples (*n* = 369) compared to normal controls (*n* = 160). (b) GOLM1 expression by HCC staging. (c) Kaplan-Meier survival analysis for GOLM1; high (*n* = 182), low (*n* = 182).

Results of different study groups have already demonstrated that GOLM1 is upregulated in HCC. Here, we conducted a brief bioinformatics analysis with GEPIA2 (http://gepia2.cancer-pku.cn/#index) using the TCGA dataset to show the expression of GOLM1 in HCC. As expected (Figure 3), GOLM1 was significantly upregulated in HCC samples compared to normal samples.

### 3.5. Mesenchymal Stem Cells Is Successfully Isolated from Human Umbilical Cords

**Figure 4:**
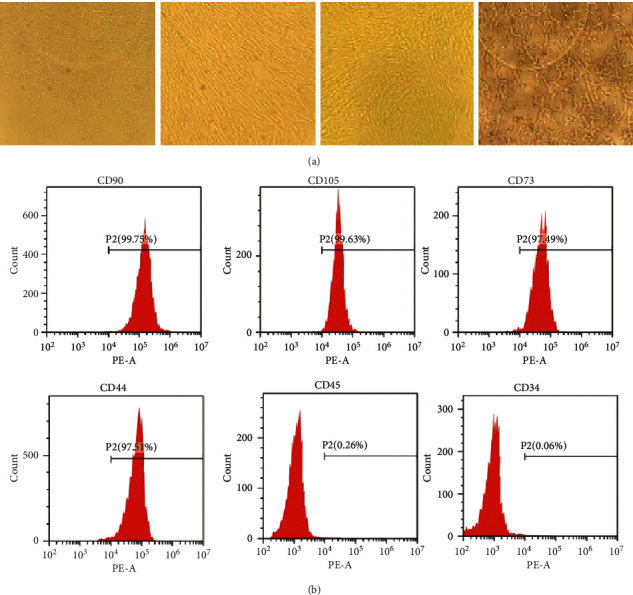
Identification of the human umbilical cord mesenchymal stem cells (hUCMSC) and hUCMSC-derived exosomes. (a) Mesenchymal stem cell morphology of the first- and second-generation umbilical cord mesenchymal stem cells (scale bar 50 *μ*m). (b) Flow cytometric analysis of the expression of cell surface markers related to mesenchymal stem cells.

Mesenchymal stem cells were successfully isolated and propagated from human umbilical cords. The cells' phenotypes were identified by determining the presence of mesenchymal stem cell markers on the surface of the cultured cells using flow cytometry (Figure 4). The markers CD 90, CD 105, CD 73, CD 44, CD 45, and CD 34 were successfully demonstrated in two independent experiments.

### 3.6. Exosomes from miR-27a-3p Transfected Mesenchymal Stem Cells

**Figure 5:**
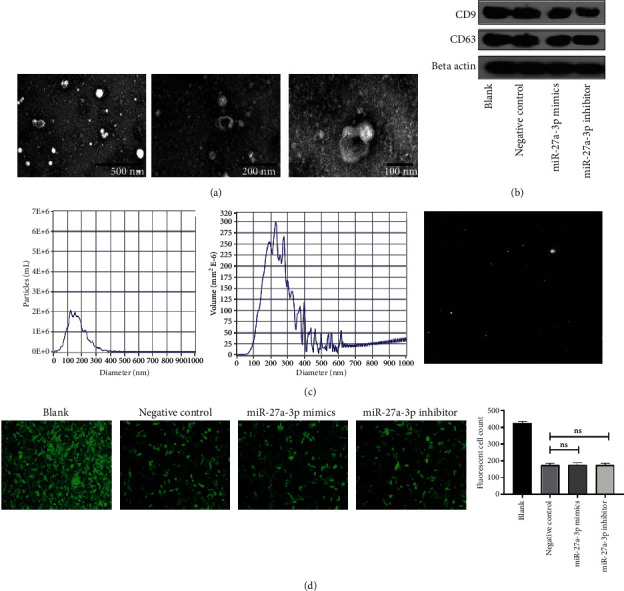
Exosomes identification. (a) Transmission electron microscopy identification of exosomes. (b) Exosome markers CD9 and CD63 confirmed by western blot. (c) Exosomes volume and purity. (d) miR-27a-3p transfection efficacy indicated by fluorescence microscopy (scale bar 50 *μ*m).

The isolated MSCs were transfected with synthesized oligonucleotides of miR-271-3p and normal controls. The cells were then cultured and exosomes isolated from the culture medium by ultracentrifugation at 100,000 g. Transmission electron microscope was used to demonstrate the structure of the exosomes (Figure 5(a)). Western blot analysis confirmed the exosomes by detecting the presence of exosome markers, CD63 and CD9 in two independent experiments (Figure 5(b)). Exosomes were further confirmed by size calculation and their purity determined (Figure 5(c)). Transfection efficacy was determined by fluorescence microscopy. Efficacy is indicated by the intensity of the green fluorescence from the fluorescence labeled miR-27a-3p (Figure 5(d)).

### 3.7. Exosomes from the miR-27a-3p Transfected Mesenchymal Stem Cells Inhibited Hepatocellular Carcinoma Proliferation

**Figure 6:**
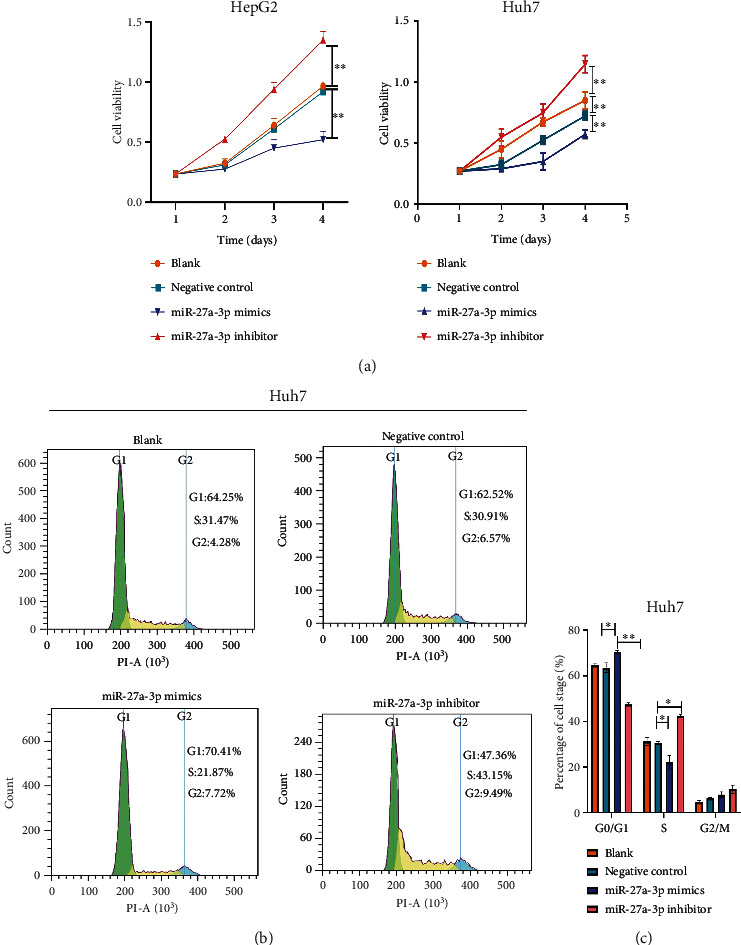
Influence of exosomal miR-27a-3p on proliferation, ability of HCC cells. (a) Exosomes containing the miR-27a-3p mimics inhibited the proliferation of Huh7 and HepG2 cells. (b, c) Exosomes containing the miR-27a-3p inhibitor significantly shortened the G0/G1 phase of HepG2 and Huh7 cells. Data were expressed as mean ± SD; ^∗^*P* < 0.05, ^∗∗^*P* < 0.01, ^∗∗∗^*P* < 0.001.

**Figure 7:**
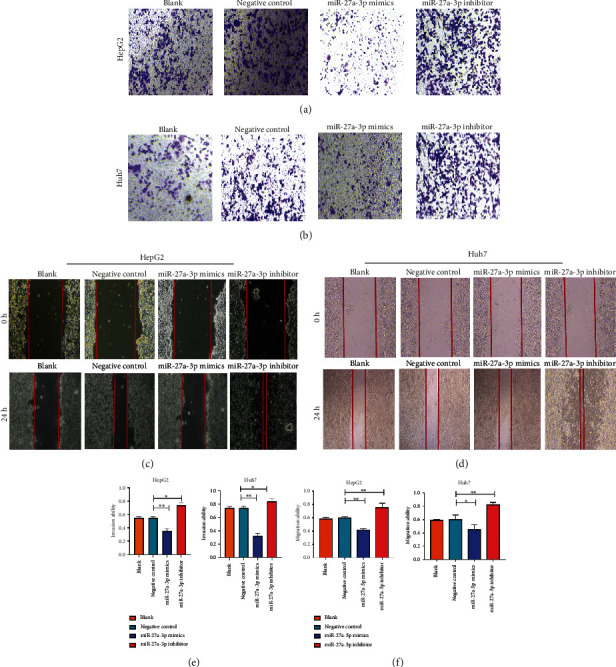
Influence of exosomal miR-27a-3p on migration and invasion ability of HCC cells. (a, b) Exosomes containing the miR-27a-3p mimics inhibited the invasion of HepG2 and Huh7 cells (scale bar 50 *μ*m). (c, d) Exosomes containing the miR-27a-3p mimics inhibited the migration of HepG2 and Huh7 cells (scale bar 50 *μ*m). Data were expressed as mean ± SD; ^∗^*P* < 0.05, ^∗∗^*P* < 0.01. (e, f) Relative quantification of the migration and invasion ability of HepG2 and Huh7 cells after treatment with exosomes containing miR-27a-3p mimics and inhibitors.

In this experiment, Huh7 and HepG2 cells were cultured and treated with exosomes extracted from the miR-27a-3p transfected MSCs. Cell proliferation, migration, and cell cycle analysis were determined using CCK-8, Transwell, and flow cytometry assays, respectively. The results showed that treatment of the cells with exosomes containing the miR-27a-3p mimics inhibited the proliferation of Huh7 and HepG2 cells (Figures 6(a) and 6(b)). Meanwhile, exosomes derived from cells containing the miR-27a-3p inhibitor did not prevent cell proliferation. The flow cytometry analysis showed that exosomes containing the miR-27a-3p inhibitor significantly shortened the G0/G1 phase of HepG2 and Huh7 cells (Figures 6(b) and 6(c)); whereas the cycle progression was significantly restrained in G0/G1 phase after cultivation with miR-27a-3p overexpressed exosomes. In the Transwell and wound healing assay, exosomes containing the miR-27a-3p mimics inhibited the migration and invasion of Huh7 and HepG2 cells while those containing the miR-27a-3p inhibitor did not prevent cell migration and invasion (Figures 7(a)–7(f)).

### 3.8. Exosomes from miR-27a-3p Transfected Mesenchymal Stem Cells Inhibited Tumor Growth, Regulated Golgi Membrane Protein 1 Expression, and Inhibited Metastasis In Vivo

**Figure 8:**
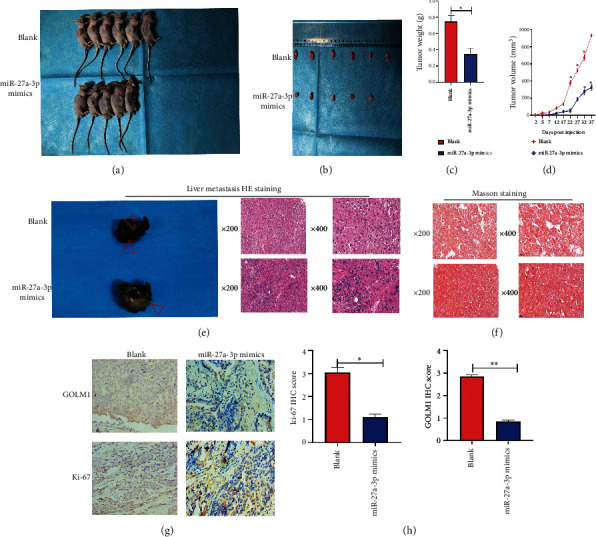
Influence of exosomal miR-27a-3p on tumor growth in vivo. (a–d) Tumors from mice injected with overexpressed miR-27a-3p containing exosomes were significantly smaller, with lower volume and weight than those from the control mice. (e–h) Results from the mouse group injected with exosomes from miR-27a-3p transfected MSCs showed inhibition of metastasis in vivo. Data were expressed as mean ± SD; ^∗^*P* < 0.05.

In vivo experiments were conducted to confirm the cell culture results. 11 balbc/nude mice were obtained and used at 8 weeks old. HepG2 cells were then multiplied in culture and injected into the mice to establish HCC models. After establishment of the tumor models, the mice were divided into two groups; one group of 5 mice were injected with exosomes extracted from miR-27a-3p transfected MSCs, while the other group of 6 mice were injected with exosomes from MSCs transfected with normal control via the tail vein. After 21 days of observation, tumors from the group of mice injected with overexpressed miR-27a-3p containing exosomes were significantly smaller, with lower volume and weight than those from the control mice (Figures 8(a)–8(d)), indicating that exosomes from miR-27a-3p transfected MSCs inhibited the progression of the HCC tumor in the mice. To demonstrate the effect of miR-27a-3p on HCC metastasis, 6 balbc/nude mice were acquired and kept as before. They were divided into two groups of 3 mice each. The mice were operated under anesthesia and cultured HepG2 cells delivered directly into the liver. In the first group, exosomes extracted from the MSCs transfected with miR-27a-3p mimics were administered directly to the liver; while in the second group, MSCs transfected with normal control was administered. The mice were followed for three weeks, sacrificed, and liver tissues collected for HE staining, Masson's staining, and immunohistochemistry. The results from the mouse group injected with exosomes from the miR-27a-3p transfected MSCs showed significantly reduced cancer spread in the liver compared to the control group, indicating that the exosomes from the miR-27a-3p transfected MSCs inhibited cancer metastasis in vivo (Figures 8(e)–8(h)).

## 4. Discussion

In this study, we were able to demonstrate that exosomes derived from umbilical mesenchymal stem cells and carrying miR-27a-3p have the ability to prevent HCC cell proliferation, migration, and metastasis both in vitro and in vivo. In the first part of this study, we demonstrated that the expression of miR-27a-3p is significantly downregulated in blood samples of HCC patients compared to healthy controls. We further showed that its expression is downregulated in both HCC cell lines and HCC tissues compared to normal liver cells and HCC paracancerous tissues, respectively, although downregulation in HepG2 and Hep3B versus LO2 were slightly smaller compared to HuH7 and MHCC97H cell lines. Furthermore, overexpression of miR-27a-3p resulted in inhibition of HCC progression, while knockdown promoted HCC progression. These findings are consistent with the results of similar studies by Li et al. [17], Yang et al. [18], and Zhao et al. [19] that all showed that miR-27a-3p is downregulated in HCC and that this downregulation plays a crucial role in promoting HCC progression, even though the exact mechanism is not known yet.

In an attempt to characterize the mechanism of action of miR-27a-3p in HCC, we utilized bioinformatics analysis and searched for HCC associated genes that are probably linked with miR-27a-3p. Four genes were retrieved, among which GOLM1 was most likely associated with miR-27a-3p after wet laboratory verification. Given that the previous experiment had shown that miR-27a-3p was most downregulated in Huh7 cell lines compared to HepG2 cells, overexpression of miR-27a-3p in Huh7 cells using miR-27a-3p-mimics resulted in downregulation of GOLM1, while its knockdown in HepG2 cells resulted in upregulation of GOLM1 expression. This confirmed that indeed miR-27a-3p downregulation most likely promotes HCC progression through regulating GOLM1 expression. miR-27a-3p belongs to the miR-27 family, which also includes miR-27b [20]. Previous studies have demonstrated the association between miR-27b and GOLM1 in HCC [21], however, this is the first study to demonstrate an association between miR-27a-3p and GOLM1 in HCC.

microRNAs are a new therapeutic target for HCC and other forms of cancer. In this context, Wang and Wu [12] rightly argued that correction of specific aberrant miRNA expression using miRNA mimics or antagomirs has the ability to normalize the affected genes and reverse cancer progression. The challenge, however, is how to safely deliver the miRNA oligonucleotides to its target gene without degradation by numerous endonucleases in the body. Fu et al. [22] extensively reviewed the major miRNA delivery systems that include, among others, modified delivery with viral vectors and nanoparticles. These modifications, although stabilize the miRNAs, have the disadvantage of triggering immunogenicity and can only load a few oligonucleotides. Exosomes and other endogenous membrane vesicles are the new methods under exploration for miRNA delivery [23–25]. They have generated a lot of enthusiasm because they are able to transfer bioactive molecules like miRNAs in a safe encapsulation that protects them from ribonucleases (RNases) in bodily fluids, while being less likely to trigger immune reactions and have negligible toxicity [22, 26]. In this study, our miR-27a-3p that was transfected in mesenchymal stem cells was able to interact with its target GOLM1 when the MSC exosomes were used to treat HCC cells. This indicated that the MSC exosomes contained in them, miR-271-3p.

Much as we attribute the success of this HCC therapeutic approach to the interaction between miR-27a-3p and GOLM1, we are also cognizant of the fact that miR-27a-3p is delivered together with other contents of mesenchymal stem cells that are known for their cell regeneration ability [27]. Various groups have researched on the use of mesenchymal stem cells to treat liver diseases such as liver fibrosis, and more recently HCC with various degrees of successes [28–30]. It is therefore likely that the effect seen could have been due to the cell regenerative ability of the mesenchymal stem cells. This needs to be verified in future experiments. Secondly, NTA analysis revealed small-sized and medium-sized vesicles ranging from 40 to 400 nm in size. Exosomes are usually from 50 to 150 nm in size. Therefore, it is likely that other small- and medium-sized vesicles could have also played a role in this result obtained.

## 5. Conclusion

In a nutshell, therefore, we have demonstrated that miR-27a-3p is downregulated in HCC and that this downregulation promotes HCC progression. We have also showed that miR-27a-3p is closely associated with GOLM1, and that overexpression of miR-27a-3p results in downregulation of GOLM1 and vice versa. We think that miR-27a-3p downregulation promotes HCC progression by upregulating the expression of GOLM1 and so overexpression of miR-27a-3p prevents HCC progression and metastasis through downregulating GOLM1. Furthermore, we were able to demonstrate that miR-27a-3p can be transfected into umbilical mesenchymal stem cells and that exosomes from the transfected cells contain miR-27a-3p that can interact with GOLM1 when treated with HCC cells.

## Figures and Tables

**Table 1:** miR-27a-3p and GAPDH primer sequences.

Name	Sequences	
miR-27a-3p	Sense	5′-TGAGGAGCAGGGCTTAGCTG-3′
	Antisense	5′-AACCACCACAGATTCACTAT-3′
GAPDH	Sense	5′GCGGAACTTAGCCACTGTGA-3
	Antisense	5′-GCAGGAGGCATTGCTGAT-3′

**Table 2:** Patient clinical characteristics.

Characteristics	miR-27a-3p		
	Patient number	Mean ± SD	*P* value
Plasma			<0.001
HCC	90	1.66 ± 0.82	
Healthy controls	75		
Gender			0.034
Male	57	1.91 ± 0.98	
Female	33	1.33 ± 1.15	
Age			0.255
<55	42	1.81 ± 1.04	
≧55	48	1.85 ± 1.21	
ALT			0.016
Negative	53	1.64 ± 1.14	
Positive	32	1.92 ± 1.03	
AST			0.001
Negative	39	1.39 ± 0.72	
Positive	51	1.40 ± 1.13	
HBV DNA (IU/mL)			0.113
<500	58	1.40 ± 0.83	
≧500	32	0.22 ± 0.71	
AFP (ng/mL)			0.879
<200	49	1.69 ± 1.05	
≧200	41	1.65 ± 1.23	
CEA (*μ*g/L)			0.677
<7.2	65	0.70 ± 1.12	
≧7.2	25	0.43 ± 1.33	
GGT (u/L)			0.707
<55	24	0.61 ± 1.18	
≧55	66	0.72 ± 1.11	
5′-NT (u/L)			0.533
<10	72	0.63 ± 1.14	
≧10	18	1.00 ± 0.62	
GLU (mmol/L)^∗^			0.047
<6.2	58	0.62 ± 1.14	
≧6.2	32	0.81 ± 1.05	

AST: aspartate aminotransferase; ALT: alanine aminotransferase; GGT: gamma glutamyl transferase; GLU: glucose; AFP: alpha fetoprotein.

**Table 3:** Association between clinical characteristics and miR-27a-3p expression level.

Parameters	Number of cases	miR-27a-3p Expression		*P*value
		Low (%)	High (%)	
Tissue				0.007
HCC	32	19	13	
Paracancerous liver	32	17	15	
Age (years)				0.123
<60	18	16	2	
≥60	14	10	4	
Gender				0.034
Male	15	13	2	
Female	17	13	4	
T stage				0.013
T1-T2	13	11	2	
T3-T4	19	15	4	
Lymph node metastasis				0.023
No	19	14	15	
Yes	13	9	14	
Differentiation				0.015
No	10	7	3	
Yes	22	19	3	
CEA grading				0.525
No	15	12	3	
Yes	17	14	3	
AFP				0.663
No	18	12	1	
Yes	14	20	5	
ALT				0.543
No	13	7	2	
Yes	19	15	4	
AST				0.592
No	19	10	5	
Yes	13	12	1	

ASL: alanine aminotransferase; AST: aspartate aminotransferase; AFP: alpha fetoprotein.

## Data Availability

All the data from this study are available upon request.

## References

[1] Sharafi H., Alavian S. M. (2019). The rising threat of hepatocellular carcinoma in the Middle East and North Africa region: results from global burden of disease study 2017. *Clinical Liver Disease*.

[2] Jemal A., Bray F., Center M. M., Ferlay J., Ward E., Forman D. (2011). Global cancer statistics. *CA: A Cancer Journal for Clinicians*.

[3] Sung H., Ferlay J., Siegel R. L. (2021). Global cancer statistics 2020: GLOBOCAN estimates of incidence and mortality worldwide for 36 cancers in 185 countries. *CA: A Cancer Journal for Clinicians*.

[4] Xu C., Zhou W., Wang Y., Qiao L. (2014). Hepatitis B virus-induced hepatocellular carcinoma. *Cancer Letters*.

[5] Liu Y., Wang J., Yang R. (2021). GP73-mediated secretion of AFP and GP73 promotes proliferation and metastasis of hepatocellular carcinoma cells. *Oncogenesis*.

[6] Ye Q. H., Zhu W. W., Zhang J. B. (2016). GOLM1 modulates EGFR/RTK cell-surface recycling to drive hepatocellular carcinoma metastasis. *Cancer Cell*.

[7] Liu Y., Zhang X., Zhou S. (2019). Knockdown of Golgi phosphoprotein 73 blocks the trafficking of matrix metalloproteinase-2 in hepatocellular carcinoma cells and inhibits cell invasion. *Journal of Cellular and Molecular Medicine*.

[8] Liu Y., Zhou S., Shi J. (2019). C-Myc transactivates GP73 and promotes metastasis of hepatocellular carcinoma cells through GP73-mediated MMP-7 trafficking in a mildly hypoxic microenvironment. *Oncogenesis*.

[9] Hou X., Yang L., Jiang X. (2019). Role of microRNA-141-3p in the progression and metastasis of hepatocellular carcinoma cell. *International Journal of Biological Macromolecules*.

[10] Xiao-Chen G. A. I., Bu-Fu T., Fang-Ming L. I. U., Yu-Ting W. U., Fang W., Hong-Bing Z. (2017). miR-27a is negatively regulated by mTOR and inhibits liver cancer cell invasion via targeting GP73. *Basic & Clinical Medicine*.

[11] Li X., Xu M., Ding L., Tang J. (2019). MiR-27a: a novel biomarker and potential therapeutic target in tumors. *Journal of Cancer*.

[12] Wang V., Wu W. (2009). MicroRNA-Based Therapeutics for Cancer. *BioDrugs*.

[13] Elsharkasy O. M., Nordin J. Z., Hagey D. W. (2020). Extracellular vesicles as drug delivery systems: why and how?. *Advanced Drug Delivery Reviews*.

[14] Arrigucci R., Bushkin Y., Radford F. (2017). FISH-flow, a protocol for the concurrent detection of mRNA and protein in single cells using fluorescence *in situ* hybridization and flow cytometry. *Nature Protocols*.

[15] Feldman A. T., Wolfe D. (2014). Tissue processing and hematoxylin and eosin staining. *Methods in Molecular Biology*.

[16] Chang J. Y. F., Kessler H. P. (2008). Masson trichrome stain helps differentiate myofibroma from smooth muscle lesions in the head and neck region. *Journal of the Formosan Medical Association*.

[17] Li J. M., Zhou J., Xu Z., Huang H. J., Chen M. J., Ji J. S. (2018). MicroRNA-27a-3p inhibits cell viability and migration through down-regulating DUSP16 in hepatocellular carcinoma. *Journal of Cellular Biochemistry*.

[18] Yang Z. F., Yang Y., Zhang R. L. (2019). Effect of microRNA-27a-3p on proliferation, apoptosis and cell cycle of hepatoma cells. *Zhonghua Gan Zang Bing Za Zhi*.

[19] Zhao N., Sun H., Sun B. (2016). miR-27a-3p suppresses tumor metastasis and VM by down-regulating VE-cadherin expression and inhibiting EMT: an essential role for Twist-1 in HCC. *Scientific Reports*.

[20] Landgraf P., Rusu M., Sheridan R. (2007). A mammalian microRNA expression atlas based on small RNA library sequencing. *Cell*.

[21] Liang H., Ai-Jun J., Ji-Zong Z. (2019). Clinicopathological significance of miR-27b targeting Golgi protein 73 in patients with hepatocellular carcinoma. *Anti-Cancer Drugs*.

[22] Fu Y., Chen J., Huang Z. (2019). Recent progress in microRNA-based delivery systems for the treatment of human disease. *ExRNA*.

[23] Robbins P. D., Morelli A. E. (2014). Regulation of immune responses by extracellular vesicles. *Nature Reviews Immunology*.

[24] Andaloussi S. E. L., Mäger I., Breakefield X. O., Wood M. J. A. (2013). Extracellular vesicles: biology and emerging therapeutic opportunities. *Nature Reviews Drug Discovery*.

[25] Wang Y., Chen X., Tian B. (2017). Nucleolin-targeted extracellular vesicles as a versatile platform for biologics delivery to breast cancer. *Theranostics*.

[26] Ha D., Yang N., Nadithe V. (2016). Exosomes as therapeutic drug carriers and delivery vehicles across biological membranes: current perspectives and future challenges. *Acta Pharmaceutica Sinica B*.

[27] Alison M., Islam S., Lim S. (2009). Stem cells in liver regeneration, fibrosis and cancer: the good, the bad and the ugly. *The Journal of Pathology*.

[28] You Y., Wen D., Gong J. P., Liu Z. J. (2019). Research status of mesenchymal stem cells in liver transplantation. *Cell Transplantation*.

[29] Hu C., Wu Z., Li L. (2020). Pre-treatments enhance the therapeutic effects of mesenchymal stem cells in liver diseases. *Journal of Cellular and Molecular Medicine*.

[30] Elkhenany H., Shekshek A., Abdel-Daim M., El-Badri N. (2019). Stem cell therapy for hepatocellular carcinoma: future perspectives. *Cell Biology and Translational Medicine*.

